# A Dual Hospital–University Specialist Training Reform to Address Workforce Shortage and Geographic Maldistribution in Indonesia: Integrative Qualitative Policy Process Review

**DOI:** 10.2196/90270

**Published:** 2026-09-09

**Authors:** Asnawi Abdullah, Yuli Farianti, Lupi Trilaksono, Arianti Anaya, Anna Kurniati, Oos Fatimah Rosyati, Etik Retno Wiyati, Mimi Sumiarsih, Christa Gumanti Manik, Nyiayu H A Sonia, Farizal Rizky Muharram, Kevin Tadeus Simanjuntak, Sydney Tjandra, Akemat Akemat, Else Mutiara Sihotang, Leni Kuswandari, Siti Yunianti, Fialisa Asriwhardani

**Affiliations:** 1 Health Policy Agency, Ministry of Health, Jl. Percetakan Negara No.29 lantai 2, RT.23/RW.7, Johar Baru, Central Jakarta City, Jakarta, 10560, Indonesia, 62 85334403303; 2 University Muhammadiyah Aceh, Banda Aceh, Indonesia; 3 Ministry of Health, Jakarta, Jakarta, Indonesia; 4 Indonesian Health Council, Jakarta, Indonesia; 5 ARC Institute Indonesia, Surabaya, Indonesia

**Keywords:** internship and residency, medical education, health workforce, health policy, health care disparities, universal health coverage, Indonesia

## Abstract

**Background:**

Indonesia faces 3 interlocking medical workforce crises: an absolute specialist deficit projected to reach 70,000 by 2032 (national density of 0.18 per 1000 population vs the Ministry of National Development Planning [Bappenas] target of 0.28), severe maldistribution (with nearly 59% of specialists concentrated in Java), and a structural anomaly in which residents pay tuition while performing essential clinical work. The 2023 Health Law (Law 17/2023) authorized a transformative reform: a hospital-based residency pathway (Rumah Sakit Pendidikan Penyelenggara Utama [primary teaching hospital; RSPPU]) operating in parallel with the long-established university-based system.

**Objective:**

This study aimed to examine the rationale, policy design, and early implementation of Indonesia’s dual hospital–university specialist medical education reform, interpreted through an 8-step change management framework developed by Kotter, and to identify transferable lessons for low- and middle-income countries.

**Methods:**

We conducted an integrative qualitative policy process review combining two evidence streams: (1) systematic documentary analysis of 19 source documents (17 primary legal, regulatory, and policy instruments plus 2 interministerial joint monitoring site-visit reports) and (2) engagement of 34 key informants through semistructured interviews and focus group discussions (45‐120 min), comprising policymakers, collegium representatives, hospital leaders, and residents across all 6 pilot sites, recruited purposively until thematic saturation. Interview and focus group data were analyzed using a hybrid deductive-inductive thematic approach with an 8-step change management framework developed by Kotter as an a priori coding frame, and member checking was completed with 7 of the 34 informants.

**Results:**

The reform designated 6 top-tier national referral hospitals as RSPPUs and enrolled 52 residents from 412 applicants (an acceptance rate of 12.6%) across 6 high-need specialties (ophthalmology, cardiology, pediatrics, orthopedics, neurology, and oncology). All 6 pilot sites established functional education units and designated institutional officials, adopted dual accreditation, and operationalized an integrated e-logbook for competency tracking, real-time monitoring of 80-hour duty limits, and anonymous bullying reporting. An interministerial joint monitoring team visited all 6 sites and scored each site as satisfactory or better across governance, curriculum, faculty, infrastructure, and learner support. Four cross-cutting themes emerged: financial-barrier removal, dual-governance pragmatism, accreditation strain, and equity-anchored deployment. Persistent tensions include variable educator compensation across hospitals; however, a standardized national framework remains under development. Mapping to the framework developed by Kotter demonstrated strong evidence for steps 1 to 6 and early evidence for steps 7 to 8.

**Conclusions:**

Indonesia’s dual hospital–university residency model is a scalable, equity-oriented, and competency-based reform that is operationally feasible and globally aligned in its early implementation. While long-term effectiveness and sustainability await longitudinal evaluation, the design offers a transferable, not yet definitively replicable, template for low- and middle-income countries confronting parallel workforce crises.

## Introduction

### The Specialist Workforce Crisis

Indonesia faces a persistent crisis in the production and equitable distribution of medical specialists, a challenge that directly undermines the quality, accessibility, and equity of health services for its 287 million people [1]. As of January 2025, national data report only 51,443 specialists, equating to a density of 0.18 specialists per 1000 population [2], significantly below the Ministry of National Development Planning (Bappenas) target of 0.28 per 1000 [3]. This overall deficit is projected to widen to 70,000 specialists by 2032 [4]. Compounding this quantitative shortage is severe maldistribution: nearly 59% of specialists are concentrated in Java and major urban centers [5], leaving vast areas of the archipelago, particularly underserved regions (Daerah Tertinggal, Perbatasan, dan Kepulauan [underdeveloped, border, and island regions; DTPK]), critically underserved [4].

This specialist deficit translates directly into significant public health vulnerability and contributes to avoidable mortality and morbidity across national priorities [6,7]. Delays in accessing obstetric and pediatric specialists contribute to persistently high maternal and neonatal mortality, particularly in regions with the lowest specialist availability [8]. The inadequacy of specialist coverage critically undermines core initiatives, including Maternal and Child Health (Kesehatan Ibu dan Anak [KIA]) programs [9]. The growing burden of noncommunicable diseases, which account for up to 73% of all deaths in Indonesia [10], necessitates timely access to specialized cardiology, oncology, and neurology services—most of which remain out of reach for populations outside major cities [9]. Low specialist density is consistently associated with adverse public health outcomes, including higher hospitalization rates for ambulatory care–sensitive conditions, longer diagnostic delays, and reduced survival for time-sensitive emergencies [11,12]. Given that the current university-based system produces only about 2700 specialists per year, eliminating the existing deficit of roughly 30,000 specialists would take at least a decade, even before accounting for retirements or the rising demand driven by population aging [5].

### Generalist and Specialist Pathways in Indonesia

To situate the present reform, we briefly describe the Indonesian medical workforce hierarchy. Generalists (dokter umum or general practitioners) complete a 5.5‐year to 6-year undergraduate medical program comprising preclinical and clinical training, followed by a 1-year mandatory national internship (Internship Dokter Indonesia) before obtaining independent licensure. As of January 2025, Indonesia had approximately 200,000 active generalists staffing community health centers (Puskesmas), district hospitals, and family practice settings [13]. Generalist training and the internship are governed by separate regulatory cascades and have been described elsewhere [14].

Specialists complete an additional residency program (Program Pendidikan Dokter Spesialis [specialist medical education program; PPDS]) lasting 2 to 6 years, in one of 39 specialties recognized by the Indonesian Health Council (Konsil Kesehatan Indonesia [KKI]) and governed by specialty collegia. Until 2023, all PPDS programs were exclusively university-based, with residents officially registered as tuition-paying postgraduate students. A consultant subspecialist tier requires a further 2 to 3 years. This paper concerns the specialist (PPDS) tier; the hospital-based RSPPU pathway introduced in 2024 sits within this tier and is the focus of analysis.

Indonesia’s existing residency framework presents a structural anomaly when benchmarked against global standards [15]. In the United States, the United Kingdom, the Netherlands, Japan, and Australia, residents are recognized as licensed members of the clinical workforce who receive salaries, benefits, and formal employment status [16-20]. This holds in Malaysia as well, which operates both hospital-based and university-based systems but pays all residents a government salary [21]. In Indonesia’s traditional university-based model, by contrast, residents are classified as tuition-paying students who pay substantial education fees while performing full clinical duties without remuneration [22]. This arrangement creates substantial equity barriers to entry, limiting the pool of aspiring physicians able to pursue specialization and constraining the size of the specialist workforce [23,24].

### The 2023 Reform: A Dual Hospital–University Pathway

To urgently address the triple crisis of insufficient production, inequitable distribution, and the structural anomaly of unpaid residency, the Indonesian government mandated a transformative reform under the 2023 Health Law [25]. This landmark legislation introduced a dual-track specialist education system that formally integrated the Hospital-Based Specialist Program (Rumah Sakit Pendidikan Penyelenggara Utama [primary teaching hospital; RSPPU]) with existing university-based programs. The new model positions designated national referral hospitals as co-organizers of residency training, ensures that residents receive living stipends and pay no tuition, and explicitly links specialist training to equity goals by binding deployment to underserved regions upon graduation. This dual approach is a comprehensive, scalable, and competency-based strategy to accelerate specialist output and drastically improve equitable access to specialized care [26].

### Aim and Research Questions

The aim of this paper is threefold: (1) to document the rationale, policy design, and early implementation of Indonesia’s dual hospital–university specialist medical education reform; (2) to interpret these processes through an 8-step change management framework developed by Kotter; and (3) to extract transferable policy lessons for low- and middle-income countries (LMICs) facing analogous workforce crises. We address three guiding research questions: (RQ1) What policy logic and political processes catalyzed the dual-track reform? (RQ2) How is the hospital-based pathway being implemented during the first 18 months and with what early outcomes? (RQ3) What systemic tensions and policy implications emerge for domestic scale-up and international adaptation?

## Methods

### Study Design and Theoretical Framework

We used an integrative qualitative policy process review design to examine the design and early operational dynamics of Indonesia’s dual-track specialist education reform. An 8-step change management framework developed by Kotter [27-29] served as both an a-priori interpretive lens and a deductive coding frame. Each step was mapped to observable indicators in the RSPPU rollout: (1) establishing urgency: quantification of the specialist deficit and high-stakes political articulation; (2) forming a guiding coalition: cross-ministerial governance arrangements involving the Ministry of Health (MoH), Ministry of Higher Education, KKI, Indonesian Accreditation Agency for Higher Education in Health (Lembaga Akreditasi Mandiri Pendidikan Tinggi Kesehatan Indonesia [LAM-PTKes]; IAAHEH), and the specialty collegia; (3) developing a change vision: Law 17/2023 and its successor regulations; (4) communicating the vision: subsequent public communication; (5) empowering broad-based action: Government Regulation 28/2024 [30], Permenkes 14/2024 [31], and Lembaga Pengelola Dana Pendidikan (Indonesia Endowment Fund for Education; LPDP) financing; (6) generating short-term wins: pilot cohort enrollment and the unified digital platform; (7) consolidating gains: joint monitoring, dual accreditation, and expansion to additional RSPPUs; and (8) anchoring change in culture: embedding mechanisms including the integrity pact, Pangkalan Data Pendidikan Tinggi (Higher Education Database; PDDikti) recognition, and the Badan Penyelenggara Jaminan Sosial (National Social Security Agency; BPJS)–linked financing pipeline.

### Study Setting and Timing of Data Collection

Data collection ran from October to November 2025, covering the first 18 months of program operation. At that point, all 52 inaugural residents were in their fourth or fifth semester; none had yet graduated, as the shortest pilot specialty (ophthalmology) requires a minimum of 7 semesters before the national board examination. The study therefore examines design and early implementation fidelity, not graduate outcomes.

### Data Sources and Triangulation

#### Documentary Analysis

The documentary corpus comprised 19 source documents: 17 primary legal, regulatory, policy, and standards documents and 2 official interministerial joint monitoring site-visit reports from December 2024 and June 2025. The 17 primary documents included Law 17/2023 on Health; Government Regulation 28/2024; Ministerial Regulations Permenkes 31/2022 and 14/2024; operational guidelines for the RSPPU program; regulatory materials from the KKI; and global and national graduate medical education standards (Accreditation Council for Graduate Medical Education [ACGME]–International [16] and IAAHEH/LAM-PTKes). Documents were retrieved between September and November 2025 from official ministerial portals. A standardized data-extraction template captured legal authority, effective date, governance roles, financing provisions, and accreditation requirements. In addition to these primary legal and regulatory instruments, we obtained and analyzed the 2 official site-visit reports produced by the interministerial joint monitoring team (December 2024 and June 2025), which applied a standardized site-assessment instrument across all 6 pilot sites; these monitoring reports formed part of the documentary evidence stream and are the source of the findings reported under “Joint Monitoring Team Findings” in the *Results* section.

#### Semistructured Key Informant Interviews and Focus Group Discussions

We purposively recruited 34 key informants spanning all institutional roles relevant to the reform (Table 1): 11 national policymakers (coded P-01 to P-11) from the MoH and the Indonesian Health Council; 7 collegium representatives (C-01 to C-07; 1-2 per specialty); 8 hospital leaders (H-01 to H-08) drawn from designated institutional officials (DIOs), Program Directors, and Clinical Competency Committees (CCCs) across the 6 RSPPU sites; and 8 enrolled residents (R-01 to R-08; 1-2 per site). Recruitment proceeded iteratively until sufficient thematic saturation was achieved across stakeholder groups.

**Table 1.** Key informant sample composition (N=34).

Stakeholder role	Informants, n	Coding scheme
National policymakers (MoH^a^, Indonesian Health Council)	11	P-01 to P-11
Collegium (1‐2 per specialty)	7	C-01 to C-07
Hospital leaders (DIOs^b^, Program Directors, and/or CCCs^c^, 1‐2 per RSPPU^d^ site)	8	H-01 to H-08
Residents (1‐2 per RSPPU site)	8	R-01 to R-08
Total	34	—^e^

a MoH: Ministry of Health.

b DIO: designated institutional official.

c CCC: Clinical Competency Committee.

d RSPPU: Rumah Sakit Pendidikan Penyelenggara Utama (primary teaching hospital).

e Not applicable.

Data were collected through semistructured key informant interviews (KIIs) and focus group discussions (FGDs). FGDs were held separately by institutional role (residents, collegium representatives, hospital leaders, and MoH technical teams) to minimize hierarchical constraints and encourage candid exchange. Several higher-level decision-makers participated in individual KIIs instead, given that their roles were less suited to group discussion. The FGD format gave participants room to respond to, clarify, and build on each other’s accounts of implementation experiences and institutional dynamics. All sessions were conducted in Bahasa Indonesia by trained qualitative researchers with prior experience in health policy and health systems research. Each session was staffed by 2 interviewers: one MoH-affiliated researcher and one external researcher. This pairing was intended to balance contextual familiarity and institutional legitimacy against independence from formal supervisory or evaluative relationships. A semistructured topic guide, structured around an 8-step framework developed by Kotter, was used throughout, supplemented by probes specific to financial mechanisms, dual accreditation, and equity-oriented deployment. Sessions lasted approximately 120 minutes, were audio-recorded with verbal informed consent, transcribed verbatim, and anonymized using a role-and-number coding scheme. Field notes were maintained throughout. Transcripts and documentary extracts were analyzed using a hybrid deductive-inductive thematic approach [32], with an 8-step framework developed by Kotter as an a-priori deductive coding frame and inductive codes capturing emergent operational tensions. Rigor was reinforced through triangulation between the documentary and interview streams, member checking of preliminary themes with 7 of the 34 informants, and an audit trail linking each theme to its source segments; reporting follows the Standards for Reporting Qualitative Research [33].

### Ethical Considerations

Verbal informed consent was obtained from all interviewees prior to recording. Data were anonymized, and any identifiable information was excluded to protect participant privacy. Formal ethical approval was not required for this study, as it involved a policy evaluation using nonclinical, nonidentifiable data [34]. No patient-level data were used.

## Results

### Overview of Findings

The integrative policy review confirms that Indonesia’s reform establishes a dual-track specialist training system that is operationally feasible and addresses critical shortages and maldistribution. Across the 2 evidence streams, four cross-cutting themes structure the findings: (1) financial barrier removal as an equity lever; (2) dual governance pragmatism; (3) accreditation strain and capacity-building; and (4) equity-anchored deployment. Triangulated evidence supports strong fidelity to Kotter’s steps 1 to 6 and early evidence for steps 7 to 8 (Table 2). Key successes include eliminating financial barriers through stipends and waived tuition, ensuring equitable access via a centralized digital selection system, and strengthening quality assurance and resident well-being through digital tools (an e-logbook with anonymous reporting). Persistent operational tensions are detailed in the dedicated subsection below.

**Table 2.** Triangulated evidence mapped to an 8-step change management framework developed by Kotter.

Kotter step	Indonesian RSPPU^a^ evidence (illustrative)	Strength of evidence
Step 1—Establish urgency	Specialist deficit 70,000 by 2032; presidential and ministerial articulation	Strong^b^
Step 2—Form a guiding coalition	Joint interministerial governance (MoH^c^, MoHE^d^, KKI^e^, IAAHEH^f^, and collegia)	Strong
Step 3—Develop vision and strategy	Law 17/2023; Government Regulation 28/2024; Permenkes 14/2024	Strong
Step 4—Communicate the vision	May 2024 Presidential launch; sustained ministerial public communication	Strong
Step 5—Empower broad-based action	LPDP^g^-financed positions; e-logbook digital platform	Strong
Step 6—Generate short-term wins	52 residents enrolled across 6 RSPPUs; 100% slots filled	Strong
Step 7—Consolidate gains	Joint monitoring (Dec 2024+Jun 2025); dual accreditation; scale-up announced	Early evidence^h^
Step 8—Anchor change in culture	Integrity pact, PDDikti^i^ integration, BPJS^j^-linked financing—all in progress	Early evidence

a RSPPU: Rumah Sakit Pendidikan Penyelenggara Utama (primary teaching hospital).

b “Strong” indicates triangulated convergence across both evidence streams.

c MoH: Ministry of Health.

d MoHE: Ministry of Higher Education.

e KKI: Konsil Kesehatan Indonesia (Indonesian Health Council).

f IAAHEH: Indonesian Accreditation Agency for Higher Education in Health (Lembaga Akreditasi Mandiri Pendidikan Tinggi Kesehatan Indonesia [LAM-PTKes]).

g LPDP: Lembaga Pengelola Dana Pendidikan (Indonesia Endowment Fund for Education).

h “Early evidence” indicates partial documentation, with mechanisms still being institutionalized.

i PDDikti: Pangkalan Data Pendidikan Tinggi (Higher Education Database).

j BPJS: Badan Penyelenggara Jaminan Sosial (National Social Security Agency).

### Documentary Findings

#### The Legal and Regulatory Cascade

##### Indonesia’s Prereform Specialist Training System

Indonesia’s specialist medical education system was historically rooted in hospital-based apprenticeship. It originated in the colonial era at the Geneeskundige Hogeschool (Medical College), where postgraduate training produced “assistant specialists” without conferring a formal specialist degree. Following independence, specialist training was re-established in the early 1950s by Universitas Indonesia and Universitas Gadjah Mada, maintaining a hospital-centered model. Admissions during this period were largely informal and managed directly by teaching hospitals [35].

A major institutional transition occurred in 1979 when the Ministry of Education and Culture assumed authority over postgraduate medical education through Decree 024/DJ/Kep/1979, shifting responsibility from the Indonesian Medical Association (Ikatan Dokter Indonesia [IDI]). In the same year, the Consortium of Health Sciences was formed to develop national guidelines and assess institutional readiness for launching residency programs [14]. Entry into residency was subsequently standardized, requiring completion of a compulsory internship and successful passage of a national competency examination, the precursor to today’s Uji Kompetensi Nasional Peserta Didik Profesi Dokter (UKNPDPD) [35].

Further refinements followed the 2004 Medical Practice Law, which established the KKI and specialty-specific collegia [35,36]. These bodies were mandated to set national training standards, oversee program accreditation, and administer board certification examinations. The system was formally codified under the 2013 Medical Education Law, which legally entrenched a university-based residency model, positioning higher-education institutions as the official organizers of specialist training in collaboration with professional associations [14]. While this framework strengthened academic oversight, it also distanced program governance from the clinical environments where most specialist training occurs, a structural tension that would later motivate the 2023 reform introducing the hospital-based RSPPU pathway.

##### From Bottleneck to Breakthrough: The 2023 Reform

Following the 2012 Higher Education Law [37] and the 2013 Medical Education Law [38], universities assumed full responsibility for curriculum design, certification, and program accreditation. While this framework was intended to safeguard academic quality, it inadvertently constrained the system’s adaptability. Annual specialist output plateaued at approximately 2700 graduates, which was insufficient to close Indonesia’s growing deficit. Stringent accreditation requirements, while essential for quality, created bottlenecks in expanding program capacity. More critically, the system classified residents as tuition-paying students despite their performance of full clinical duties [22], imposing financial hardship and contradicting global norms in which residents are recognized as supervised professionals and compensated accordingly.

Prior to the introduction of the RSPPU model, the MoH pursued multiple strategies to alleviate Indonesia’s chronic specialist shortage: (1) expanding enrollment quotas in university-based residency programs; (2) implementing the Wajib Kerja Dokter Spesialis (mandatory specialist service program; WKDS) mandatory rural service program, later invalidated by the Supreme Court [33]; (3) establishing academic health system networks to integrate universities, teaching hospitals, and local governments; and (4) sponsoring physicians for specialist training abroad. While each measure yielded partial gains, none addressed the systemic constraints of a rigid, university-centric training architecture. Both the scale-up of training capacity and the retention of graduates in underserved regions remained elusive.

**Figure 1.**
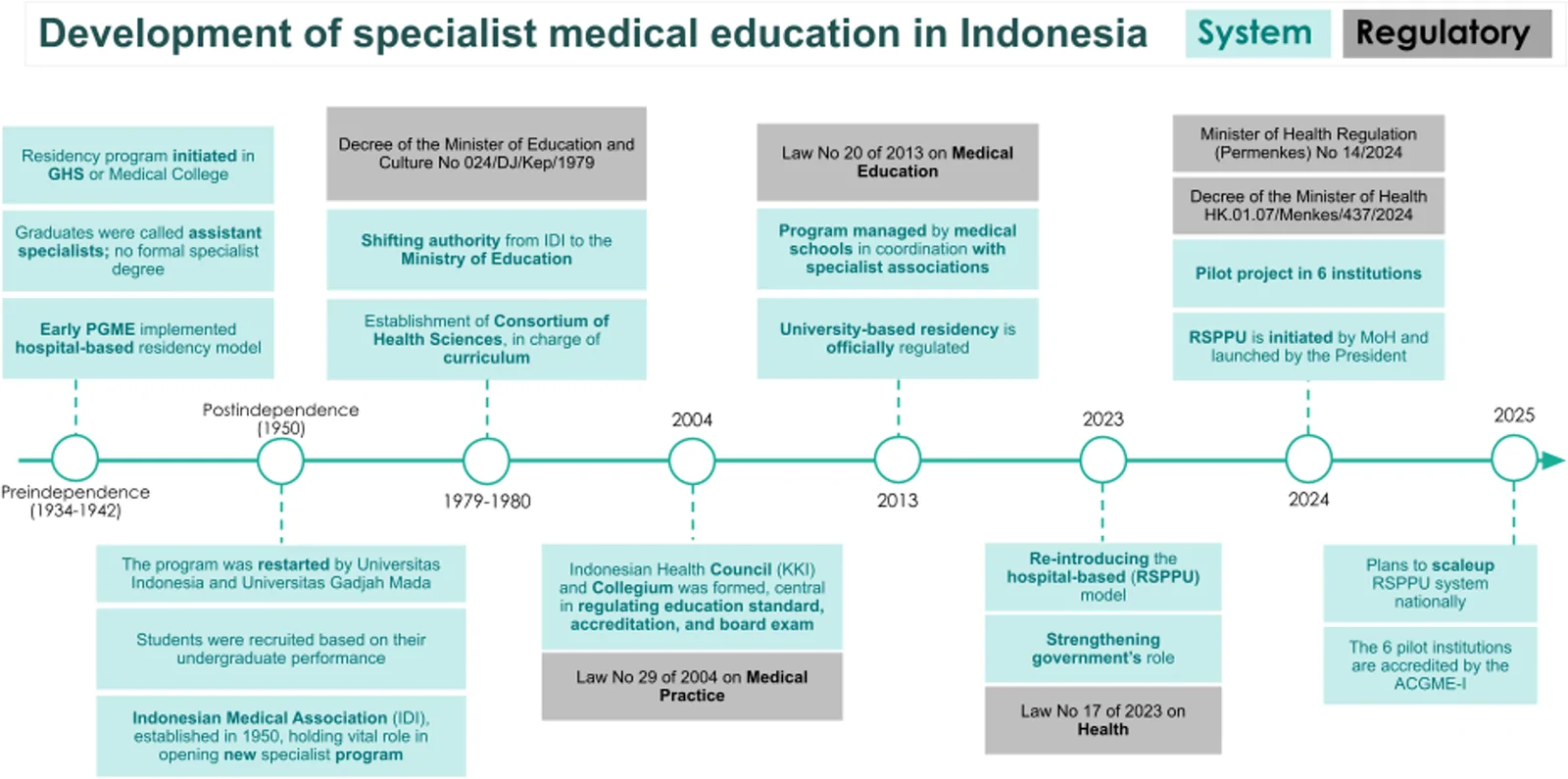
Chronological development of specialist medical education in Indonesia, from the colonial-era Geneeskundige Hogeschool through the 2023 hospital-based Rumah Sakit Pendidikan Penyelenggara Utama (primary teaching hospital; RSPPU) pathway. The lower text block now reads: “Hospital-based specialist training was re-established (‘restarted’) under the RSPPU model in 2024 after a 45-year hiatus during which all specialist training had been university-led.” [14,35]. ACGME-I: Accreditation Council for Graduate Medical Education–International; GHS: Geneeskundige Hogeschool (now Faculty of Medicine, Universitas Indonesia); MoH: Ministry of Health; PGME: Postgraduate Medical Education.

By 2022, Indonesia’s chronic specialist shortage—exacerbated by a double burden of disease and a rapidly aging population—had reached a tipping point. Policymakers proposed a structural recalibration: revival of a hospital-based residency model, informed by international experience in the United Kingdom, the United States, South Korea, and Singapore. The approach did not seek to replace universities but to complement them, recognizing that the vast majority of specialist training already occurs in clinical settings. With nearly 500 hospitals formally affiliated with medical schools as teaching sites and over 3000 hospitals nationwide, the untapped institutional capacity was substantial. This vision was codified in Health Law 17/2023, which formally introduced the RSPPU, primary teaching hospitals authorized to serve as co-organizers of specialist training. The proposal triggered significant interinstitutional tension. Universities expressed concern about diminished academic authority, while the Ministry of Higher Education raised questions about degree legitimacy and quality assurance. After months of deliberation, a pragmatic compromise was forged: RSPPUs would assume primary responsibility for training delivery, mentorship, and day-to-day program management, while universities would retain cosignatory authority over professional degrees and academic certification. The operational parameters were detailed in Government Regulation No 28/2024 and in MoH Regulation 14/2024. The development of specialist medical education in Indonesia is summarized chronologically in Figure 1.

### Pilot Cohort Profile and Selection Outcomes

The inaugural cohort comprised 52 residents selected from 412 (overall acceptance rate 12.6%) eligible applicants nationally. All 52 announced positions were filled. Distribution across pilot specialties was as follows: ophthalmology (Cicendo Eye Hospital, n=8 slots filled, from 96 applicants); cardiology (National Cardiovascular Center Hospital Harapan Kita, n=10, from 87 applicants); pediatrics (National Women and Children Health Center Hospital Harapan Kita, n=8, from 79 applicants); orthopedics (Prof Dr Soeharso Orthopedic Hospital, n=10, from 64 applicants); neurology (National Brain Center Hospital, n=10, from 45 applicants); and oncology (Dharmais Cancer Hospital, n=6, from 41 applicants). As of December 2025, no resident had withdrawn; all 52 remained enrolled.

### Joint Monitoring Team Findings

The joint monitoring exercise was documented in 2 official interministerial site-visit reports that were analyzed as part of the documentary evidence stream (see *Methods*). A joint monitoring team composed of representatives from the MoH (Directorate of Health Human Resources Provision), the Ministry of Higher Education, IAAHEH/LAM-PTKes, and the relevant specialty collegia conducted 2 structured site visits: the first in December 2024 (approximately 7 mo after the May 2024 presidential launch) and the second in June 2025 (approximately 13 mo post-launch). At each visit, a standardized 23-item instrument adapted from the ACGME-International Self-Study framework assessed five governance domains: (1) governance and DIO leadership; (2) curriculum implementation and milestone tracking; (3) faculty development and supervision ratios; (4) infrastructure (simulation, library, and e-logbook uptime); and (5) learner support (well-being, anonymous reporting use, duty-hour compliance). Each domain was scored on a 4-point ordinal scale (excellent, satisfactory, needs improvement, or unsatisfactory). Across both visits, all 6 pilot sites were rated satisfactory or better on all 5 domains, with no domain at any site scored needs improvement or unsatisfactory (Table 2, Kotter step 7).

### Implementation: Hospital Selection, Governance, and Training Processes

#### Hospital Designation and Functional Education Units

**Figure 2.**
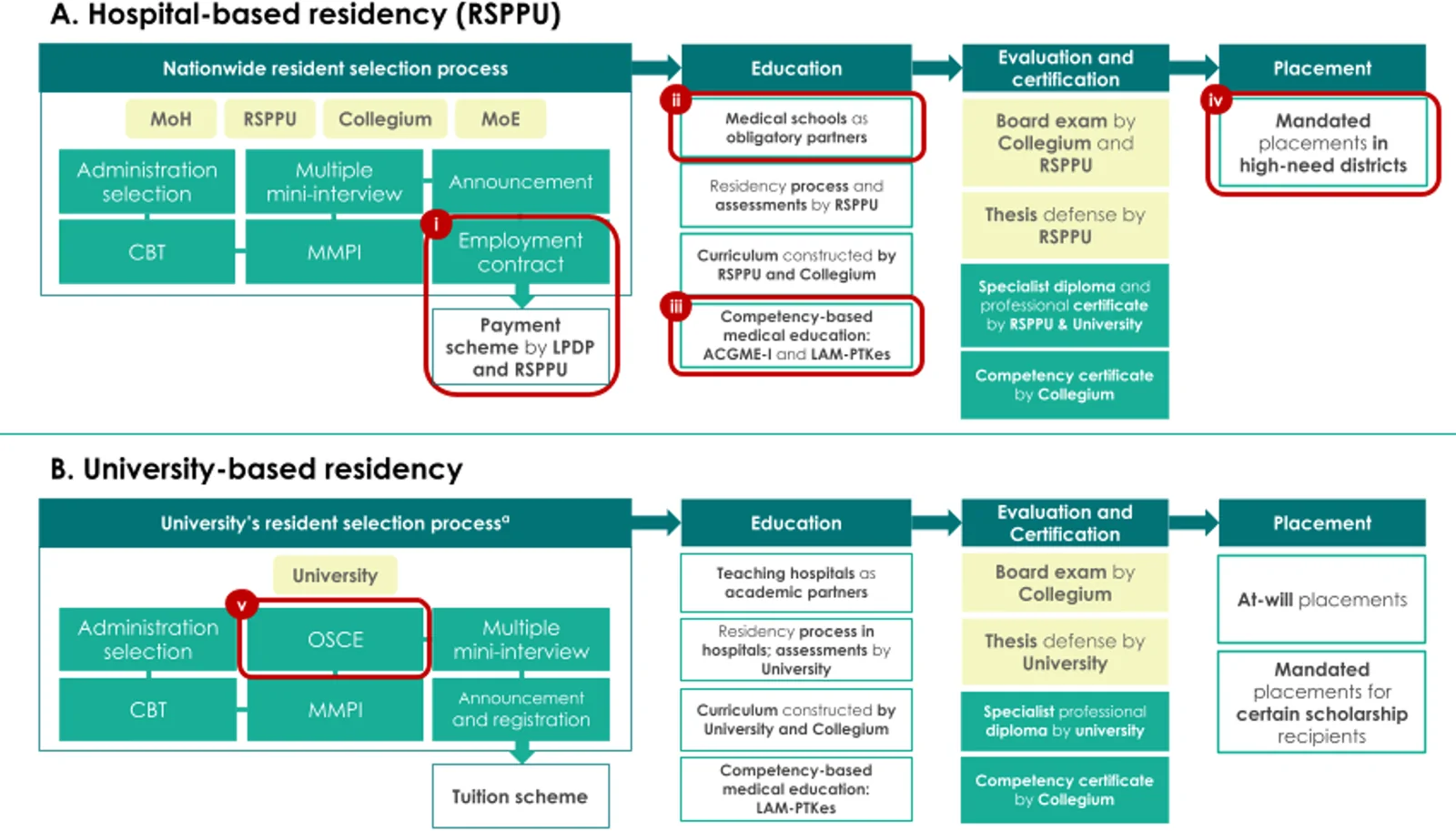
Comparative process flow for university-based vs hospital-based specialist training. Red highlight boxes mark distinctive Rumah Sakit Pendidikan Penyelenggara Utama (primary teaching hospital; RSPPU)–specific elements: (A) the binding preadmission integrity pact for return-of-service; (B) the LPDP-financed full tuition and stipend coverage; and (C) the dual Accreditation Council for Graduate Medical Education–International (ACGME-I)/Indonesian Accreditation Agency for Higher Education in Health (Lembaga Akreditasi Mandiri Pendidikan Tinggi Kesehatan Indonesia [LAM-PTKes]; IAAHEH) accreditation requirement. *Process may vary by university regulation and autonomy. CBT: Computer-Based Test; CCC: Clinical Competency Committee; DIO: designated institutional official; LPDP: Lembaga Pengelola Dana Pendidikan (Indonesia Endowment Fund for Education); MMPI: Minnesota Multiphasic Personality Inventory; MoE: Ministry of Education; MoH: Ministry of Health; OSCE: Objective Structured Clinical Examination; PDDikti: Pangkalan Data Pendidikan Tinggi (Higher Education Database); PEC: Program Evaluation Committee.

By mid-2024, the reform transitioned from policy to practice, with the designation of 6 national referral hospitals as pilot RSPPUs (Figure 2). Hospitals were selected based on demonstrable institutional commitment, top-tier (Paripurna) accreditation, high clinical caseloads, and robust infrastructure. Although all 6 had previously served as affiliate training sites or hosted fellowship programs, none functioned as primary teaching hospitals under the university-based model. This historical positioning afforded them greater autonomy to design residency curricula fully embedded within their clinical ecosystems. Each RSPPU established a functional education unit responsible for curriculum delivery, mentorship, and quality assurance, with duty hours capped at 80 hours per week and night shifts integrated into a structured schedule. Crucially, residents are now formally recognized not as tuition-paying students but as supervised health professionals entitled to social protection, welfare safeguards, and employment-based rights—a fundamental shift codified in Permenkes 14/2024.

#### Dual Accreditation and Curriculum Codevelopment

To align with global best practices, the MoH adopted core elements of the ACGME-International framework. Each RSPPU appointed a DIO responsible for graduate medical education oversight, governance, and regulatory compliance. While hospitals initially struggled to meet the ACGME-I expectation that DIOs dedicate 80% of their time to educational duties, given their concurrent clinical responsibilities, ACGME-led training workshops proved instrumental in clarifying foundational governance concepts, including the CCC and Program Evaluation Committee (PEC). Concurrently, all RSPPU programs remain subject to national accreditation by IAAHEH/LAM-PTKes. To ensure parity with university-based residencies, both tracks adhere to the same core curriculum, as defined by the National Specialist Education Standard issued by KKI. Program-specific adaptations (muatan lokal) are permitted to reflect institutional strengths; for example, Cicendo Eye Hospital integrates community ophthalmology as a distinguishing component.

#### Selection Process, Financing, and Graduation Pathway

Registration was conducted via a unified online information system accessible nationwide. Every applicant signed an integrity pact pledging to return to their region of origin upon graduation. The selection protocol incorporated administrative screening, computer-based cognitive testing, psychological assessment (including the Minnesota Multiphasic Personality Inventory [MMPI]), and a structured interview. Successful candidates received an employment contract rather than a tuition invoice. The e-logbook supported structured documentation of clinical procedures, milestone-based competencies, real-time duty-hour monitoring, and anonymous reporting of bullying or mistreatment. All program-related costs were fully covered by the LPDP. In 2024, the Ministry of Finance allocated 1000 funded positions with a target of 5000 specialists within 2 years. Upon completion, residents undergo a dual evaluation: a national board examination administered by the collegium and the RSPPU and a thesis defense overseen by the hospital. Graduates receive a joint specialist diploma cosigned by the RSPPU and the partner university and a national competency certificate issued by the collegium. The final phase—mandatory deployment to districts with critical shortages of specialists—operationalizes the reform’s redistributive mission.

### Stakeholder Themes From KIIs

#### Theme 1: Financial Barrier Removal as an Equity Lever

All 8 residents interviewed (R01 to R08) emphasized that the elimination of tuition fees and the introduction of a monthly stipend were the single decisive enabler of their decision to pursue specialization, particularly for those from outside Java:

At that time, I was not financially able to pay for specialist training on my own, so when support from the Ministry of Health became available, especially because it was structured as a scholarship, it further strengthened my motivation to pursue specialist training.

[R-08, Pediatrics]

#### Theme 2: Dual Governance: Pragmatism Over Purism

National policymakers (P-01 through P-04) and a representative of the Indonesian Health Council framed the reform as a functional realignment rather than an institutional displacement:

Until today, some still see RSPPU as competition. It is not. University-based and hospital-based training must coexist. We share the same vision: to ensure Papua and Maluku have their own specialists.

[P-07, Indonesian Health Council]

#### Theme 3: Accreditation Strain and Capacity-Building

Hospital leaders and collegium representatives described initial challenges in reconciling ACGME-International expectations with national IAAHEH standards, mainly highlighting documentation requirements and navigating interim governance structures. Meanwhile, the complementarity in quality was also valued; intensive capacity-building measures were implemented to ensure quality clinical teaching.

But if we look closely, both (IAAHEH and ACGME) essentially emphasize quality aspects, and ACGME further reinforces this.

[C-05, Collegium representative]

We examined 2 accreditation frameworks: the ACGME, and, like it or not, LAM-PTKes as well.

[H-03, RSPPU pilot]

#### Theme 4: Equity-Anchored Deployment

Policymakers and university administrators emphasized the structural placement of equity at the point of entry, not exit:

Selection in the hospital-based program is national, transparent, and system-based. All stages—written test, psychological test, interview—are computerized and involve the Ministry of Health, Higher Education, hospitals, and the professional collegia. Subjectivity is minimal.

[P-06, then Directorate of Health Human Resources Provision]

#### Faculty and University Administrator Perspectives

The DIO is expected to dedicate eighty percent of their work to education and only twenty percent to clinical service. In reality, however, our primary role is fundamentally service-oriented, as we are functionally appointed for clinical care. This has become one of the practical challenges we face in the field: how to divide our time appropriately.

[H-07, Senior Consultant Faculty, Radiation Oncology]

#### Early Implementation Tensions and Resident Experience

Three implementation tensions remained unresolved at 18 months. First, residents reported uncertainty about their formal recognition in the PDDikti national student database, a status that affects access to certain academic benefits such as government scholarships for subspecialization, library privileges, and postgraduate rail and air fare discounts. As of February 2026, an interministerial working group had agreed in principle on a dual learner-employee status entry, but technical integration was still pending. Second, the first selection round (May 2024) exposed logistical inequities: of 412 applicants, 301 (73.1%) came from outside Java, yet all centralized written and computer-based examinations required travel and accommodation that applicants estimated at IDR 4 to 9 million (US $250‐$570). Third, no national framework exists to compensate clinical educators for the additional teaching workload. One pilot RSPPU introduced interim honoraria arrangements, but the variation across sites raises equity concerns and is not sustainable at scale.

### Mapping Findings to an 8-Step Framework Developed by Kotter

Triangulated evidence mapped to an 8-step framework developed by Kotter showed strong fidelity for steps 1 to 6 and early, partial evidence for steps 7 to 8 (Table 2). Step 1 (urgency) was established through the 70,000-specialist deficit projection and high-level political articulation. Step 2 (guiding coalition) is operationalized through the joint interministerial monitoring team. Step 3 (vision) is codified in Law 17/2023. Step 4 (communication) was executed through the nationally broadcast May 2024 presidential launch and sustained public communication by the MoH. Step 5 (empowerment) is reflected in successor regulations (Government Regulation 28/2024, Permenkes 14/2024) and LPDP financing. Step 6 (short-term wins) is evidenced by the enrollment of 52 residents and the operational unified digital platform. Step 7 (consolidating gains) is partially evidenced through dual accreditation and announced expansion to additional RSPPUs in 2025 to 2026. Step 8 (anchoring in culture) remains incomplete: the integrity pact, PDDikti integration, and BPJS-linked financing each represent unfinished embedding mechanisms.

## Discussion

### Principal Findings

This integrative policy process review of Indonesia’s dual-track specialist training reform yields 3 principal findings. First, the RSPPU model is operationally feasible at 18 months: all 6 pilot hospitals established the requisite educational infrastructure, all 52 selected residents remain enrolled, and a multiagency joint monitoring framework documented satisfactory or better performance across governance, curriculum, faculty, infrastructure, and learner support. Second, the reform’s defining innovation is structural rather than pedagogical. By abolishing resident tuition, reclassifying residents as supervised health professionals, and binding selection to an equity-anchored integrity pact, the model recasts specialist training as a public investment in spatial health equity rather than a private financial transaction. Third, the persistent implementation tensions, including dual-accreditation adaptation, the absence of a national clinical-educator compensation framework, PDDikti integration delays, and Java-centric examination logistics, are operational and tractable rather than conceptual and define a clear policy agenda for scale-up.

### Comparison With Prior Work

The RSPPU model corrects a longstanding misalignment: specialist training has always taken place predominantly in hospitals, yet formal authority resided with universities. The 2023 Health Law resolves this not through institutional substitution but through functional realignment. Hospitals now lead curriculum delivery, mentorship, and day-to-day program management; universities retain cosignatory authority over degrees; and professional collegia ensure that both pathways adhere to the National Specialist Education Standard [39]. Internationally, Indonesia’s design sits between fully hospital-employer models such as the United Kingdom NHS and the United States ACGME, and university-mandated systems found in parts of Continental Europe and Japan. Malaysia’s parallel dual system [21] is the closest structural comparator, but Indonesia’s model is distinctive in 3 respects: the explicit binding of admission to a return-of-service integrity pact; LPDP-financed full coverage of resident fees; and a dual ACGME-I/IAAHEH accreditation requirement applied to every pilot site. Unlike the US model, Indonesia’s pathway emerged from negotiated tripartite compromise rather than a top-down mandate. Unlike the UK’s centrally employed model, it preserves academic coauthority. This hybridity may be what makes the model relevant to other LMICs whose health and higher-education ministries operate in parallel rather than in aligned hierarchies.

### Operationalization of Equity

The reform’s equity imperative is operationalized through 3 reinforcing mechanisms. First, a unified national selection platform removes geographic barriers, allowing physicians from Aceh to Papua to compete on equal terms. Second, a binding integrity pact at the point of entry obliges graduates to serve in their regions of origin, a commitment built into admission rather than imposed after graduation. Third, residents are now salaried professionals rather than fee-paying students. With tuition fully covered by LPDP and monthly stipends provided, the model removes the largest financial barrier that historically excluded talented but underresourced candidates, bringing Indonesia into line with the global norm of recognizing residents as members of the clinical workforce. The equity mechanism operates at entry, not exit. Prior postgraduation deployment schemes such as the WKDS were judicially invalidated [40], and exit-based coercion has failed in multiple LMICs. By embedding the return-of-service obligation in the admission contract and financing the residency with public funds, the reform converts deployment from a contested instrument of coercion into a consensual condition of participation.

### Early Implementation Considerations

Early implementation across 6 national referral hospitals confirms operational feasibility. The joint monitoring framework, itself a Kotter step 7 consolidation mechanism, produced satisfactory or better ratings in both the 2024 and 2025 site visits. Several tensions identified in the *Results* nonetheless require interpretation. The variability in clinical-educator compensation across pilot sites is not a temporary inconvenience but a fundamental design gap. Without a national framework, scale-up risks producing a 2-tier system in which residents at well-resourced flagship hospitals receive structured mentorship while those at expanded RSPPUs encounter underincentivized faculty. The PDDikti integration delay signals a related institutional question: are RSPPU residents learners, employees, or both? Resolving this status ambiguity will require coordinated action by the MoH and the Ministry of Higher Education, ideally through a dual-status entry in PDDikti that preserves academic benefits while reflecting the residents’ employment-grade compensation. From a Kotter perspective, these tensions are characteristic of steps 7 and 8. The reform has generated visible wins and structural commitments, but cultural and bureaucratic embedding will require sustained leadership attention through at least the first graduating cohort in 2028.

### Policy Implications

The reform’s domestic durability hinges on five concrete actions: (1) issuance of a national clinical-educator compensation framework anchored in BPJS Health case-mix reimbursement, with a minimum hourly teaching credit codified in a Permenkes successor regulation; (2) full PDDikti integration with an explicit dual learner-employee status to resolve resident benefit ambiguity; (3) regional decentralization of selection examination hubs to neutralize Java-centric travel costs; (4) statutory consolidation of the integrity-pact return-of-service obligation into the new specialist license cycle so that it is enforceable through licensure renewal; and (5) a national tracking platform for RSPPU graduates’ geographic deployment, retention beyond the initial bond, and downstream clinical outcomes in destination districts.

Four design lessons are transferable to LMICs facing the same triple workforce challenge of undersupply, maldistribution, and structurally unpaid residency, though direct replication is not implied. *(*1) Anchor reform in primary legislation, not subordinate regulation alone. Indonesia’s Law 17/2023 created the political foundation under which negotiated compromises could be operationalized through successor regulations. Lower legal instruments are reversible by a single minister. (2) Resolve the resident-status anomaly first. Removing financial barriers is the largest single equity lever and the most politically defensible against reversal. (3) Build dual accreditation deliberately. Treating ACGME-I and a strong national accreditor as complementary rather than competing reduces foreign-perception risk while preserving sovereignty. (4) Engineer equity at entry, not at graduation. A binding integrity pact at admission is administratively simpler and politically more durable than postgraduation deployment schemes, which have failed in multiple LMICs including Indonesia’s own invalidated WKDS [40] and earlier Malaysian iterations [21].

### Strengths and Limitations

#### Strengths

The integrative design captures policy-process complexity unavailable in single-method studies; member checking with 7 of 34 informants and triangulation across documentary and interview streams reduce interpretive bias; Kotter operationalization provides an auditable analytic frame; and reporting follows the Standards for Reporting Qualitative Research [33].

#### Limitations

Four limitations warrant acknowledgment. First, the study covers only the first 18 months of program operation; effectiveness on graduate output, geographic retention, and clinical outcomes will require longitudinal cohort tracking. A separate study has been initiated and is expected to report in 2028. Second, residents’ lived experience is captured at a single early point; structured longitudinal follow-up is planned. Third, this is a single-country case, and cross-LMIC comparative evaluation is needed to test transferability claims empirically. Fourth, while triangulation was applied, some members of the authoring team are professionally affiliated with the reform’s implementation.

### Conclusions

Indonesia’s dual hospital-university residency model addresses the nation’s specialist shortage and geographic maldistribution through structural rather than incremental reform. By designating national referral hospitals as co-organizers of training under collegium-led standards, the RSPPU pathway eliminates tuition barriers, provides living stipends, and directs deployment to underserved regions through a binding integrity pact. Early implementation across 6 pilot sites demonstrates operational feasibility: residents are salaried professionals trained in high-volume clinical settings, supported by a digital platform for transparent selection, competency tracking, workload monitoring, and anonymous well-being reporting. Remaining challenges, including accreditation adaptation, educator incentives, learner-database integration, and centralized examination logistics, are operational and tractable rather than conceptual.

Pending longitudinal evaluation of graduate output, geographic retention, and clinical outcomes, this model offers a transferable design template for LMICs confronting parallel workforce crises, though it is not yet a definitively replicable blueprint. Whether the reform delivers on its equity promise will be answered not by its design but by whether the first 52 residents and the cohorts that follow practice in the districts where Indonesia’s health system most needs them.

## Abbreviations

ACGME-I: Accreditation Council for Graduate Medical Education–International
BPJS: Badan Penyelenggara Jaminan Sosial (National Social Security Agency)
CCC: Clinical Competency Committee
DIO: designated institutional official
DTPK: Daerah Tertinggal, Perbatasan, dan Kepulauan (underdeveloped, border, and island regions)
FGD: focus group discussion
IAAHEH: Indonesian Accreditation Agency for Higher Education in Health (Lembaga Akreditasi Mandiri Pendidikan Tinggi Kesehatan Indonesia [LAM-PTKes])
IDI: Ikatan Dokter Indonesia
KII: key informant interview
KKI: Konsil Kesehatan Indonesia (Indonesian Health Council)
LMIC: low- and middle-income country
LPDP: Lembaga Pengelola Dana Pendidikan (Indonesia Endowment Fund for Education)
MMPI: Minnesota Multiphasic Personality Inventory
MoH: Ministry of Health
PDDikti: Pangkalan Data Pendidikan Tinggi (Higher Education Database)
PEC: Program Evaluation Committee
PPDS: Program Pendidikan Dokter Spesialis (specialist medical education program)
RSPPU: Rumah Sakit Pendidikan Penyelenggara Utama (primary teaching hospital)
UKNPDPD: Uji Kompetensi Nasional Peserta Didik Profesi Dokter (national competency examination)
WKDS: Wajib Kerja Dokter Spesialis (mandatory specialist service program)
